# Severe Hypertriglyceridemia in a Patient With Metabolic Syndrome and Psoriasis on Risankizumab-Rzaa

**DOI:** 10.1210/jcemcr/luae087

**Published:** 2024-06-21

**Authors:** Isra Abdulwadood, Jose F De melo, Robert C Scheel, John P Bois

**Affiliations:** Alix School of Medicine, Mayo Clinic, Scottsdale, AZ 85259, USA; Department of Internal Medicine, Mayo Clinic, Rochester, MN 55905, USA; Department of Internal Medicine, Mayo Clinic, Rochester, MN 55905, USA; Department of Cardiovascular Medicine, Mayo Clinic, Rochester, MN 55905, USA

**Keywords:** hypertriglyceridemia, hyperlipidemia, hyperviscosity syndrome, chylomicronemia syndrome, risankizumab-Rzaa, plasmapheresis

## Abstract

We report a case of severe hypertriglyceridemia (HTG) complicated by hyperviscosity syndrome as a possible adverse reaction to risankizumab-rzaa in a 49-year-old male with a history of longstanding uncontrolled type 2 diabetes, obesity, and coronary artery disease with prior ST-elevation myocardial infarction. On admission, the patient presented with xanthomatous plaques, chest and epigastric discomfort, and headache. Subsequent blood testing revealed severely elevated triglyceride (TG) levels at 7670 mg/dL (86.59 mmol/L) [reference range: <150 mg/dL; 1.69 mmol/L] and total cholesterol at 934 mg/dL (24.14 mmol/L) [reference range: <200 mg/dL; 5.17 mmol/L]. Triglyceride levels decreased and symptoms resolved with dietary restrictions and plasmapheresis. At follow-up, his TG remained elevated but improved, and he was advised to continue lipid-lowering medications as well as cessation of risankizumab. While the patient presented with high risk factors, we posit that the subacute presentation of severe HTG is a possible result of his recent course of risankizumab-rzaa therapy for management of psoriasis. This is noteworthy as pharmaceutical surveys and clinical trials do not list severe HTG as an adverse effect. Postmarketing surveillance studies are essential to confirm this potential association and monitor drug safety. In summary, this case highlights a possible link between risankizumab and severe HTG, emphasizing the importance of ongoing pharmacovigilance to identify and manage unexpected adverse effects associated with new medications.

## Introduction

Hypertriglyceridemia (HTG) is 1 of the most common lipid abnormalities encountered in clinical practice (1). In most patients, HTG arises from a combination of multiple genetic variations and environmental risk factors. Contrarily, in this article we present the case of a patient who presented with HTG and subsequent hyperviscosity syndrome, raising concern for a severe side effect from a recently introduced monoclonal antibody, risankizumab-rzaa, used in the treatment of psoriasis.

## Case Presentation

**Figure 1.**
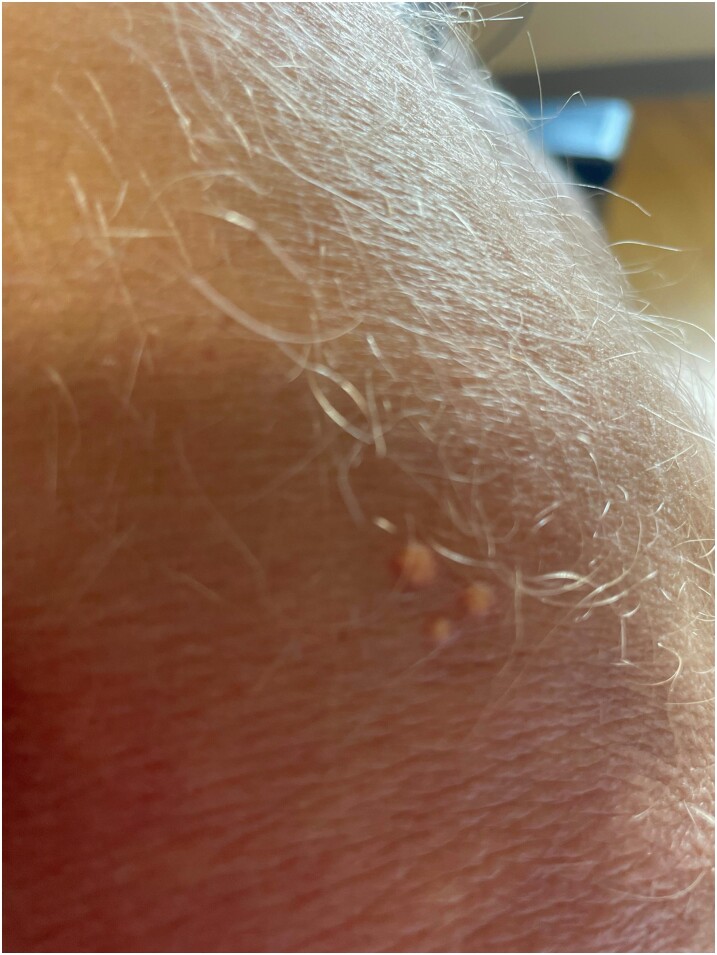
Xanthomatous lesions visualized on right posterior forearm.

A 49-year-old male with relevant medical history of truncal obesity with a body mass index of 31 kg/m^2^, current smoking with a 40-pack year history, hypertension, longstanding uncontrolled type 2 diabetes mellitus (T2DM), coronary artery disease with prior ST-elevation myocardial infarction requiring coronary angioplasty 2 years prior, and psoriasis presented to the cardiology clinic of this hospital for evaluation of severe HTG. The patient was in his usual state of health until 8 weeks prior to his presentation when he developed small, white plaques on his back, posterior forearms, lower extremities, and gluteal region, which were consistent with xanthomas (Fig. 1). Five weeks later, the patient experienced occasional headaches, chest and epigastric pain, as well as diffuse allodynia and skin hypersensitivity. His medications included metformin, glimepiride, lisinopril, high-intensity atorvastatin for management of elevated cardiovascular risk, and risankizumab, which he had received subcutaneously every 90 days for the last 2 years. His last injection had been approximately 1 month prior to his presentation. His hemoglobin A1c was 8.7% (95.09 mmol/mol) [reference range: <5.7%; 38.8 mmol/mol]. The average TG levels ranged from 200 to 500 mg/dL (2.26 mmol/L to 5.64 mmol/L) [reference range: <150 mg/dL; 1.69 mmol/L] in the two years that preceded his presentation.

## Diagnostic Assessment

Laboratory evaluation obtained on admission showed severely elevated total cholesterol levels at 934 mg/dL (24.14 mmol/L) [reference range: <200 mg/dL; 5.17 mmol/L] and TG level of 7670 mg/dL (86.59 mmol/L) [reference range: <150 mg/dL; 1.69 mmol/L]. It is important to note that our laboratory was unable to accurately count values greater than 800 mg/dL (9.032 mmol/L); therefore, the low-density lipoprotein and high-density lipoprotein cholesterol values were unable to be calculated. Serum lipase was also elevated at 138 U/L (2.30 µkat/L) [reference range: 13-60 U/L; .22-1 µkat/L], but cross-sectional imaging of the abdomen showed no evidence of acute pancreatitis. Liver and biliary enzymes including alanine aminotransferase, aspartate aminotransferase, and alkaline phosphatase were normal. An electrocardiogram demonstrated normal sinus rhythm with nonspecific ST and T wave abnormalities but no significant change from the previous report. All relevant laboratory tests can be found in Table 1. Given markedly elevated TG accompanied by neurologic symptoms and chest pain, he was diagnosed with hyperviscosity syndrome.

**Table 1.** Laboratory values

	Initial Visit value (SI unit)	1 month Visit value (SI unit)	3 month value (SI unit)	Reference range (SI unit)	Comment
CBC					Manually checked, lipemic
Hemoglobin, g/dL	12.6 g/dL (126 g/L)			13.2-16.6 g/dL (132-166 g/L)	
Hematocrit, %	36.5 (36.5)			38.3-48.6 (38.3-48.6)	
Erythrocytes, ×10(12)/L	38.3-48.6 ×10(12)/L (38.3-48.6 ×10^12^/L)			4.35-5.65 ×10(12)/L (4.35-5.65 ×10^12^/L)	
Platelet count, ×10(9)/L	278 ×10(9)/L (278 ×10^9^/L)			135-317 ×10(9)/L (135-317 ×10^9^/L)	
White blood cell count, ×10(9)/L	12.2 ×10(9)/L (12.2 ×10^9^/L)			3.4-9.6 ×10(9)/L (3.4-9.6 ×10^9^/L)	
General chemistry					Visible lipemia, centrifuged prior to analysis
Sodium, mEq/L	130 mEq/L (130 mmol/L)			135-145 mEq/L (135-145 mmol/L)	
Potassium, mEq/L	5.2 mEq/L (5.2 mmol/L)			3.6-5.2 mEq/L (3.6-5.2 mmol/L)	
Chloride, mEq/L	96 mEq/L (96 mmol/L)			98-107 mEq/L (98-107 mmol/L)	
Bicarbonate, mEq/L	20 mEq/L (20 mmol/L)			22-29 mEq/L (22-29 mmol/L)	
BUN, mg/L	210 mg/L (21 mg/dL)			80-240 mg/L (8-24 mg/dL)	
Creatinine, mg/L	12.6 mg/L (1.26 mg/dL)			7.4-23.5 mg/L (.74-2.35 mg/dL)	
eGFR*^a^,* mL/min/BSA	70 mL/min/BSA (70 mL/min*^b^*)			≥ 60 mL/min/BSA (≥ 60 mL/min*^b^*)	
Glucose, mg/dL	378 mg/dL (20.97 mmol/L)			70-140 mg/dL (3.89-7.78 mmol/L)	
Total protein, g/dL	5.9 g/dL (59 g/L)			6.3-7.9 g/dL (63-79 g/L)	
Lactate, mg/dL	75.56 mg/dL (4.2 mmol/L)			9.01-39.64 mg/dL (.5-2.2 mmol/L)	
Lipase, U/L	138 U/L (2.30 µkat/L)			13-60 U/L (.22-1 µkat/L)	
Lipids					
Total cholesterol, mg/dL	934 mg/dL (24.14 mmol/L)	215 mg/dL (5.57 mmol/L)	151 mg/dL (3.90 mmol/L)	< 200 mg/dL (5.17 mmol/L)	
Triglycerides, mg/dL	7670 mg/dL (86.59 mmol/L)	1421 mg/dL (16.05 mmol/L)	609 mg/dL (6.87 mmol/L)	<150 mg/dL (1.69 mmol/L)	Calculated value is not valid over 800 mg/dL
LDL cholesterol, mg/dL	—	—	40 mg/dL (1.03 mmol/L)	<100 mg/dL (2.59 mmol/L)	Triglyceride value > 800 mg/dL, calculated LDL cholesterol is not valid
HDL cholesterol, mg/dL	—	22 mg/dL (.57 mmol/L)	25 mg/dL (.65 mmol/L)	≥ 40 mg/dL (1.03 mmol/L)	Specimen was lipemic, result cancelled by ancillary
Non-HDL cholesterol, mg/dL	—	—	126 mg/dL (3.26 mmol/L)	< 130 mg/dL (3.36 mmol/L)	Unable to quantitate, result cancelled by ancillary
Thyroid					
TSH, µg/L	.0028 µg/L (2.8 mIU/L)			.0003–.0042 µg/L (.3-4.2 mIU/L)	Visible lipemia, centrifuged prior to analysis

Abbreviations: BSA, body surface area; BUN, blood urea nitrogen; CBC, complete blood count; eGFR, estimated glomerular filtration rate; HDL, high-density lipoprotein; LDL, low-density lipoprotein.

^*a*^eGFR calculated using the 2021 CKD_EPI creatinine equation.

^*b*^eGFR without normalization to BSA.

## Treatment

**Figure 2.**
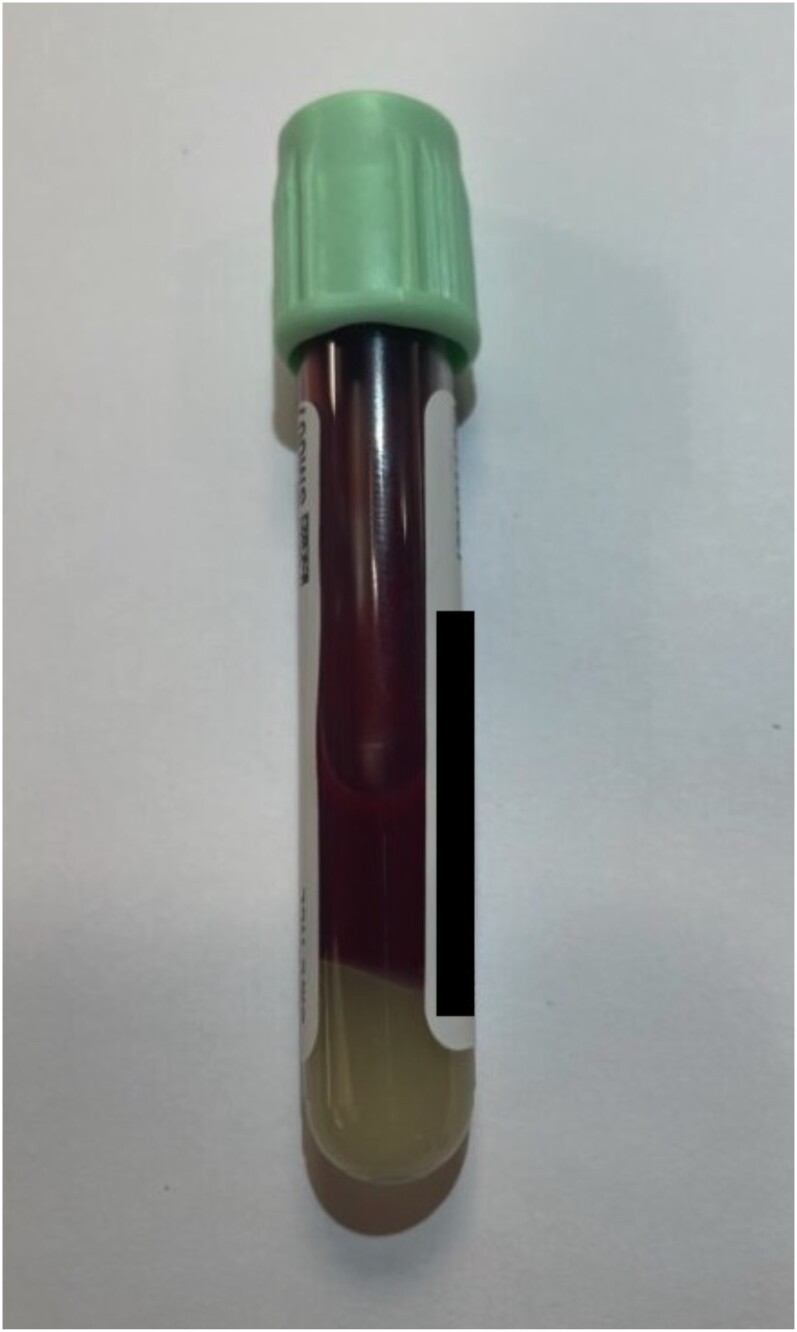
Initial hematologic specimen collected.

Following hospital admission, the patient was placed on a “nothing by mouth” diet, intravenous insulin was started, and he underwent a total of 2 plasma exchange sessions on hospital days 2 and 3 (Fig. 2). Further plasma exchange treatments were not pursued as the patient experienced an allergic reaction (manifesting as urticaria and periorbital edema) after the second session, prompting administration of intravenous antihistamine medication. TG downtrended to 2601 mg/dL (29.38 mmol/L) on hospital day 2 after the first session of plasma exchange and to 885 mg/dL (9.98 mmol/L) after the second treatment, with subsequent resolution of his neurologic symptoms. He was transitioned to a basal-bolus insulin regimen on hospital day 4, and a fat- and carbohydrate-restricted diet was initiated. He continued high-intensity statin therapy, and fenofibrate and icosapent ethyl (omega 3 fatty acids) were started for chronic management of HTG.

## Outcome and Follow-up

Following discontinuation of plasma exchange therapy, TG levels stabilized at 1500 mg/dL (16.94 mmol/L) on hospital day 5, so he was discharged in improved conditions with strict dietary recommendations. He was also advised to discontinue risankizumab and consult his dermatologist for an alternative psoriasis management plan.

At his 1-month follow-up, the patient reported no recurrence of his initial symptoms, improvement of xanthomas, and adherence to antilipid therapy with no major side effects. The lipid panel was repeated and showed total cholesterol of 215 mg/dL (5.55 mmol/L) and TG of 1421 mg/dL (16.05 mmol/L) (Table 1). Low-density lipoprotein was not calculated. Injectable glucagon-like peptide 1 agonist was recommended for management of diabetes, obesity, and HTG [though he did not incorporate liraglutide .6 mg/.1 mL injection (18 mg/3 mL) until 5 months after hospitalization due to difficulty with insurance coverage]. One week later, on repeat blood tests, his TG levels decreased to 282 mg/dL (3.18 mmol/L), which was within the goal of <500 mg/dL (5.65 mmol/L). The patient was advised to continue current dietary recommendations and medication regime of a daily high-dose rosuvastatin 40 mg, daily fenofibrate 145 mg, and twice-daily omega 3 fatty acid 2 mg.

At his 3-month follow-up, the patient continued to deny recurrence of his initial symptoms. His lipid panel resulted in a total cholesterol of 151 mg/dL (3.90 mmol/L) and an increase in TG to 609 mg/dL (6.88 mmol/L) (Table 1). Moving forward, the patient was recommended to resume dual antiplatelet therapy (clopidogrel 75 mg and aspirin 81 mg) until TG were controlled and to return for follow-up with labs in 6 months.

## Discussion

Severe HTG is defined as TG levels over 1000 mg/dL (2). Common causes of severe HTG include primary disorders, genetically based, or secondary disorders, nongenetically based (2). Primary disorders, not recognized in the patient presented here, commonly present as early as infancy as chylomicronemia syndromes (CS) and include lipoprotein lipase (LPL) deficiency or apolipoprotein (Apo) C-II deficiency (3). Secondary disorders include metabolic conditions such as diabetes, obesity, alcohol overconsumption, chronic kidney disease, and, more rarely, hypothyroidism (2, 3). The prevalence of diabetes, more specifically uncontrolled or poorly controlled T2DM, has been reported in up to 76% of patients with severe HTG and is thought to be exacerbated by the combination of LPL deficiency and continued increased dietary fat intake (4). Commonly prescribed medications have also been associated with severe HTG such as thiazide diuretics, beta blockers, estrogen, corticosteroids, antipsychotics and antidepressants, and even protease inhibitors through a variety of known and unknown mechanisms (3). Most notably, immunosuppressants such as sirolimus and everolimus, mammalian target of rapamycin inhibitors administered as chemotherapeutic agents, have been shown to increase TG levels via an increase in Apo C-III levels and inhibition of LPL (5). Rarely, severe HTG may also develop due to autoimmune hyperlipidemia or with glycogen storage disease type 1 (3).

Severe HTG may result in complications such as acute pancreatitis, fatty liver disease, cardiovascular disease, and hyperviscosity syndrome (2). Hyperviscosity syndrome, as diagnosed in the case here, describes an abnormally elevated blood viscosity due to a pathological overproduction of blood components including elevated levels of TG (2). This can lead to a range of symptoms and complications, including neurologic symptoms such as headache, fatigue, and an increased risk of blood clot formation. We suspect HTG complicated by hyperviscosity syndrome manifested as headaches and chest pain in the patient presented here.

Management of HTG is largely directed at preventing severe complications. The approach algorithm includes identification and subsequent removal of aggravating factors such as uncontrolled diabetes, alcohol excess, or medications that can be safely discontinued or replaced. Patients may also be advised to manage weight and reduce fat intake. Finally, pharmacotherapy in the form of high-dose fibrates, omega-3 fatty acids, niacin, and statins should be initiated simultaneously (3). TG-lowering agents such as icosapent ethyl (Vascepa) and more novel agents such as Apo C-III and angiopoietin-like protein 3 modulators have emerged as key regulators of TG metabolism, though such therapies are less readily available or currently under investigation (6, 7). Furthermore, therapeutic plasmapheresis is not a mainstay of severe HTG management, more commonly utilized for the management of acute pancreatitis; however, it has been reported to successfully lower TG levels in patients with recurrent pancreatitis and in pregnant patients with familial CS (8).

As described in the case here, the patient did not present with primary, genetically based disorders but did present with the risk factors of truncal obesity and longstanding uncontrolled T2DM. Nevertheless, we posit that his presenting TG level is disproportionately elevated when compared to his metabolic risk factors. It is therefore possible that his severe HTG and hyperviscosity syndrome represented an adverse effect (AE) of risankizumab therapy, as it was the only discernable change in the patient's recent medical history. While the literature affirms that severe HTG or CS may be a result of monogenic or polygenic forms of LPL deficiency/insufficiency (3), based on his age at presentation, we predict genetics likely played a limited role in his disease presentation, though genetic screening is being considered.

Risankizumab-rzaa, a humanized IgG1 monoclonal antibody that inhibits interleukin 23A, was approved for the treatment of moderate to severe plaque psoriasis, psoriatic arthritis, and Crohn's disease in 2022 (9, 10). To our knowledge, no drug survey or clinical trial performed prior to risankizumab approval includes severe HTG or CS as a possible side effect; however, the literature suggests that drugs, including immunomodulators, may act as aggravating factors (3). Understanding the specific mechanism would require further research; however, having been only recently introduced to the market, the drug currently lacks comprehensive data on long-term safety and possible AEs. A real-world postmarketing pharmacovigilance analysis published in 2023 by Shu et al reported 79 significant AEs with the most frequently reported being COVID-19, pneumonia, pruritus, cerebrovascular accident, and myocardial infarction (9). Further surveillance studies will be necessary to confirm our suspicion of risankizumab therapy resulting in the possible adverse effect of severe HTG.

In summary, we describe a case of severe HTG and hyperviscosity syndrome in a 49-year-old male with a history of longstanding uncontrolled T2DM, truncal obesity, and coronary artery disease. This case highlights a possible link between risankizumab therapy and severe HTG, emphasizing the importance of ongoing pharmacovigilance to identify and manage unexpected long-term AEs associated with new medications.

## Learning Points

- Risankizumab-rzaa is an effective therapy for plaque psoriasis via interleukin-23A inhibition, and reports of serious side effects are rare.
- We present a case of HTG complicated by hyperviscosity syndrome as a potential adverse effect of risankizumab-rzaa therapy, which increases the risk for acute pancreatitis and coronary artery disease.
- Hyperviscosity syndrome describes an abnormally elevated blood viscosity due to a pathological overproduction of blood components that can lead to neurologic complications and an increased risk of blood clots.
- Managing severe HTG requires good control of diabetes and dietary lipid levels, though plasmapheresis may be considered for symptomatic patients.

## Data Availability

Data sharing is not applicable to this article as no datasets were generated or analyzed during the current study.
